# Photonic amorphous topological insulator

**DOI:** 10.1038/s41377-020-00368-7

**Published:** 2020-07-24

**Authors:** Peiheng Zhou, Gui-Geng Liu, Xin Ren, Yihao Yang, Haoran Xue, Lei Bi, Longjiang Deng, Yidong Chong, Baile Zhang

**Affiliations:** 1 National Engineering Research Center of Electromagnetic Radiation Control Materials, University of Electronic Science and Technology of China, 610054 Chengdu, China; 2 Division of Physics and Applied Physics, School of Physical and Mathematical Sciences, Nanyang Technological University, 21 Nanyang Link, Singapore, 637371 Singapore; 3 Centre for Disruptive Photonic Technologies, The Photonics Institute, Nanyang Technological University, 50 Nanyang Avenue, Singapore, 639798 Singapore

**Keywords:** Quantum optics, Photonic crystals, Quantum optics, Photonic crystals

## Abstract

The current understanding of topological insulators and their classical wave analogs, such as photonic topological insulators, is mainly based on topological band theory. However, standard band theory does not apply to amorphous phases of matter, which are formed by non-crystalline lattices with no long-range positional order but only short-range order, exhibiting unique phenomena such as the glass-to-liquid transition. Here, we experimentally investigate amorphous variants of a Chern number-based photonic topological insulator. By tuning the disorder strength in the lattice, we demonstrate that photonic topological edge states can persist into the amorphous regime prior to the glass-to-liquid transition. After the transition to a liquid-like lattice configuration, the signatures of topological edge states disappear. This interplay between topology and short-range order in amorphous lattices paves the way for new classes of non-crystalline topological photonic bandgap materials.

## Photonics: finding one-way pathways in unlikely places

Optical circuits that provide high-speed data traffic even in the presence of unexpected defects can now be fabricated from materials with less than perfect crystallinity. In photonic topological insulators, interactions between crystal energy bands produce unique ‘edge states’ that enable photons to move in one direction. Yidong Chong and Baile Zhang from Nanyang Technological University in Singapore and colleagues have investigated how disorder impacts the formation of these edge states. The team placed gyromagnetic cylindrical rods into a waveguide and arranged them in patterns ranging from periodic to completely unstructured. Probing the waveguide with microwave radiation revealed that short-range interactions enabled the one-way states to persist in amorphous structures until the lattice transformed into a liquid-like structure. The amorphous edge states proved robust enough to pass photons around rectangular obstacles and across large air gaps.

## Introduction

Photonic topological insulators (PTIs)^1–5^ are an emerging class of photonic bandgap materials that can impart “topological protection” to photons, in the same way topological insulator materials do for electrons. The most striking feature enabled by topological protection is the existence of edge states that are protected against perturbations and defects, for which several promising applications have been identified, including robust lasers^6–8^ and robust optical delay lines^9^. Topological protection originates from the topology of the underlying photonic band structures. The most basic class of topological insulators, Chern insulators, have integer band invariants called Chern numbers that are computed using Bloch band states, which in turn owe their existence to the discrete translational symmetry of the lattice^10–13^. Consequently, the vast majority of PTIs have been based on periodic lattices^2,13–24^ such as photonic crystals, which possess both long-range and short-range positional order. Long-range order is connected to the lattice periodicity, while short-range order is related to the regular connectivity of neighboring sites throughout the lattice^25^.

In addition to photonic crystals, there are many photonic materials without periodicity, such as photonic quasicrystals^26–28^ and photonic amorphous materials^29–31^. It is therefore natural to ask whether topological edge states can exist in these photonic materials. Some authors have pointed out that photonic topological edge states can exist even in the absence of lattice periodicity^32–34^. Of course, any local disorder can be regarded as breaking translational periodicity in the underlying lattice, and topological edge states are protected against weak disorder (for a sufficiently strong disorder, topological protection breaks down^32^). More surprisingly, certain lattices are topologically trivial in the absence of disorder but become “topological Anderson insulators” when disorder is added, as recently demonstrated using a photonic lattice^34^. In discrete systems (e.g., tight-binding models), the Bott index was shown to be usable in place of the Chern number when there is no well-defined momentum space^33,35^. The aforementioned scenarios all start from a periodic lattice with a well-defined Brillouin zone, into which local disorder is introduced.

There are many materials in nature that exist in amorphous phases (e.g., glass, polymers, and gels) that lack any such easily identifiable “initial” crystalline configuration. Amorphous phases of matter intrinsically lack long-range order but maintain short-range order^36^. They exhibit an interesting phenomenon known as the “glass transition”, whereby an amorphous medium experiences a dramatic structural change from a glass-like phase into a liquid-like phase. To date, many physical aspects of the glass transition remain poorly understood^37^.

To study the interplay between band topology and short-range order, we have experimentally extended a Chern number-based PTI^10–13^ into the amorphous regime. The resulting topological insulating phases in amorphous lattices are also known as amorphous topological insulators^38–41^. Similar to previous theoretical proposals^42,43^, the amorphous PTI that we study consists of gyromagnetic rods that are arranged in computer-generated amorphous lattice patterns and magnetically biased to break time-reversal symmetry. By performing edge/bulk transmission and near-field distribution measurements, we experimentally verify the existence of robust topological edge states in the amorphous PTIs prior to the onset of the glass transition. When the lattice undergoes the glass transition, the local site connectivity is dramatically altered, resulting in the closing of the bulk topological gap and the disappearance of the topological edge states. Although the concept of amorphous topological insulators has been theoretically proposed in condensed matter systems^38–40^ and some related features have been realized in a mechanical network of gyroscopic oscillators^41^, there has never been any systematic experimental study of how band topological effects depend on short-range order (including the important role of the glass transition). This work thus enriches our understanding of topological photonic materials and paves the way for exploration of new types of photonic lattices that can host topologically protected edge states.

## Results

**Fig. 1:**
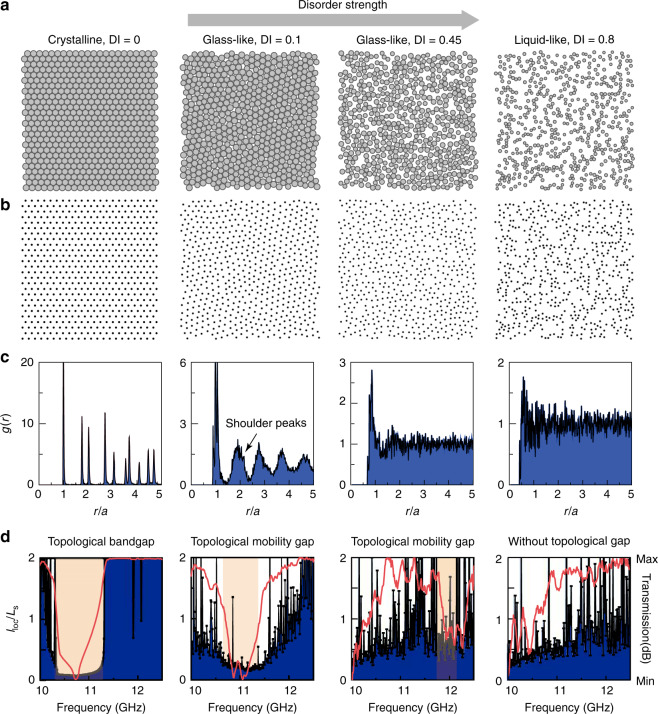
Transition of photonic lattices with increasing disorder. **a**, **b** Particle patterns **a** and the corresponding photonic lattices **b** with different structural correlations. The DI = 0 case is a triangular lattice. The glass-like lattices with strong short-range order have DI = 0.1 and 0.45. The liquid-like lattice with weak short-range order possesses DI = 0.8. **c** Pair correlation function *g*(*r*) for the different lattices. **d** Numerically calculated localization lengths (black curves) and bulk transmission (red curves) for the photonic lattices. The orange regions are the frequency windows in which topological edge states can be observed. The calculation details can be found in the Supplementary Information Note 3

Photonic lattices with different structural correlations are generated using particle packing methods previously developed in soft condensed matter physics^44,45^. The packing is conducted in a two-dimensional (2D) square unit cell with periodic boundary conditions and bidisperse discs (radius ratio of 1.2 with equal distributions; see Supplementary Information Note 1). The process ends upon reaching a target packing density *ϕ*, defined as the filling ratio of discs. The packing density *ϕ* is inversely related to the structural disorder—the lower *ϕ* is, the more disorder. As shown in Fig. 1a, the maximum packing density *ϕ*_max_ (=0.9069) corresponds to the crystalline triangular lattice without disorder. We define a disorder index (DI) as DI = (*ϕ*_max _− *ϕ*)/*ϕ*_max_, such that the DI is positively related to the disorder strength—the higher the DI is, the more disorder. Note that the particle packing algorithm is used simply to generate an abstract lattice configuration; after the lattice is generated, the finite discs can be replaced with sizeless dots (Fig. 1b). In the generated abstract lattice configurations, the lattice sites will be occupied physically by identical gyromagnetic rods. At this stage, we will scale the sizes of the generated lattice configurations to maintain a constant density of gyromagnetic rods as a reference for comparison among different samples. This scaling has no effect on the structural disorder.

Here, we examine the pair correlation function

1 $$g(r) = \frac{{a^2}}{{4N\pi r^2}}\mathop {\sum}\limits_{i = 1}^N {\mathop {\sum}\limits_{j = 1}^N {\left\langle {\delta \left( {r - r_{ij}} \right)} \right\rangle } }$$

where *r* is the distance between a pair of gyromagnetic rods, *N* is the number of rods, *δ* is the delta function, *<>* means the ensemble average, and $a = L/\sqrt N$ (*L* is the size of the lattice system). This function quantifies the degree of structural correlation in the lattice and has been extensively employed to characterize amorphous phases^46^. The pair correlation functions for different lattice structures are plotted in Fig. 1c. For DI = 0, *g*(*r*) shows sharp peaks in the whole *r* range. For DI = 0.1, the first peak in *g*(*r*) splits into subpeaks due to bidisperse packing; the shoulders of the second peak indicate local clustering, a common phenomenon in amorphous materials^46^; and the other peaks progressively damp away at *r*/*a* > 3, indicating the lack of long-range order. For DI = 0.45, the short-range order decreases, and the first *g*(*r*) peak is less than half of its counterpart in the DI = 0.1 case. For the weakly correlated lattice with DI = 0.8, there is only one visible peak, and *g*(*r*) ~ 1 over most of the range, indicating weak short-range order (i.e., a liquid-like lattice configuration).

These variations in lattice properties can have significant impacts on band topological phenomena. In the following content, we will start with the topological bandgap of a crystalline PTI corresponding to a DI = 0 lattice (left panel in Fig. 1d). We will demonstrate that this topological gap persists for amorphous lattices up to DI = 0.45 (middle panels in Fig. 1d) before the glass transition. After the glass transition, the topological frequency gap closes (right panel in Fig. 1d).

**Fig. 2:**
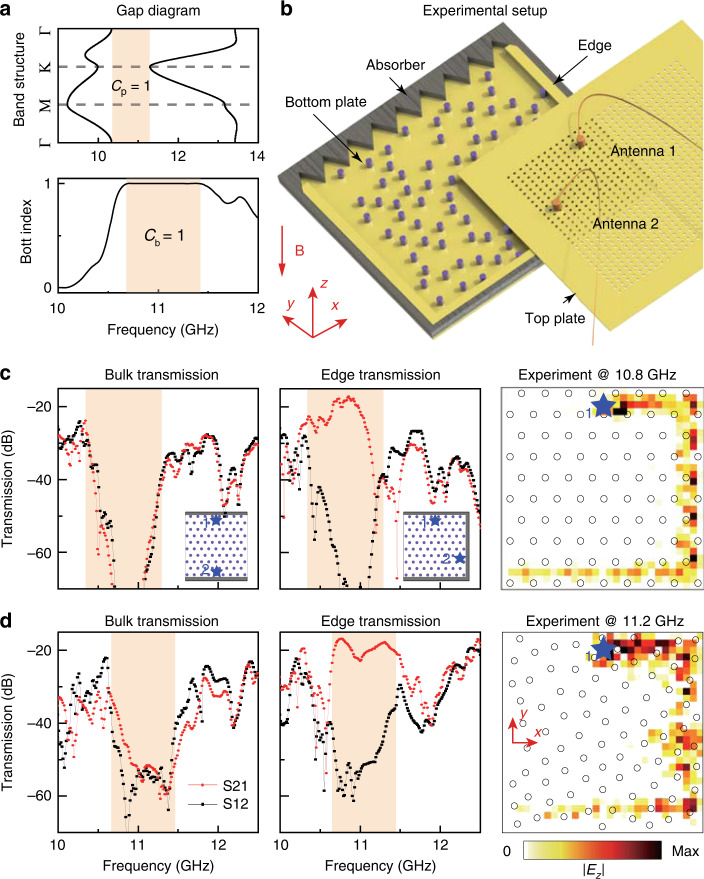
Observation of topological states in an amorphous PTI. **a** Numerically calculated band structure of the crystalline PTI (upper panel, DI = 0) and Bott index of the amorphous lattice (lower panel, DI = 0.1). The orange regions denote the frequency windows corresponding to the topological gaps. **b** Schematic of the experimental setup. The top plate contains cylindrical holes with a radius of 1 mm. The probe and source dipole antennas (1 and 2) are inserted into the waveguide through these holes. Three sides of the waveguide are wrapped with metal walls acting as perfect electric conductor (PEC) boundaries. The other side is covered by microwave absorbers. **c** Measured S21 and S12 transmissions of bulk and edge states and |*E*_*z*_| field distribution in the crystalline PTI. Insets: schematics of the experimental setup showing the photonic lattice (purple dots) and metal boundaries (gray bars) for the bulk and edge measurements. The source (1) and probe (2) antennas are indicated by blue stars. **d** Measured S21 and S12 transmissions for bulk and edge states and |*E*_*z*_| field distribution in the amorphous PTI

The crystalline PTI corresponds to a triangular lattice with the lattice constant *a*_c_ = 18.81 mm, with gyromagnetic rods having a radius of 2.2 mm. (See Supplementary Information Note 1 for detailed system parameters.) The band structure is shown in Fig. 2a: there is a bandgap between the second and third transverse magnetic (TM) bands (note that the first band is not shown). From the Bloch functions, we compute the gap Chern number to be *C*_p_ = 1^43^.

To characterize the present photonic lattice, as well as the others, we use the experimental setup shown in Fig. 2b. The sample lies in a copper parallel plate waveguide. Gyromagnetic ferrite cylindrical rods are placed on the bottom plate, and an external static magnetic field of *B* = 0.2 T is applied along the negative *z* direction. The sizes of all samples are tailored to be 9*a* × 9*a* to fit our apparatus. The parallel plate waveguide has a spacing of 4 mm, which supports only the fundamental TM mode below 37.5 GHz. To facilitate the excitation and measurement of electromagnetic fields inside the waveguide, a square array of air holes is drilled through the top plate. As the diameter of these holes is smaller than 1/15 of the operational wavelength, they have a negligible influence on the electromagnetic modes in the waveguide, as verified via first-principles calculations (see Supplementary Information Note 2).

To study the bulk states, source and probe antennas are placed near the top and bottom edges (near the metal walls), while the left and right sides of the photonic lattice are wrapped with microwave absorbers (Fig. 2c). The measured forward (S21) and backward (S12) transmission through the bulk shows a dip from 10.4 GHz to 11.3 GHz, indicating a bandgap^47^, consistent with the simulated results shown in Fig. 1d. For the edge state measurement, three sides of the lattice are wrapped with metal walls, and the remaining side is covered with absorbers. In this way, only clockwise (anticlockwise) propagation excited at point 1 (2) can be detected. The measured edge transmissions show a very large difference between forward and backward transport in the gap, indicating the existence of topological one-way edge states. We also map out the field distributions inside the waveguide, showing how the unidirectional edge state travels through two 90° sharp corners without reflection (it is then absorbed after impinging on the microwave absorber). Due to the energy leakage from the imperfect contacts of the experimental assembly, the small radiation loss of the holes drilled in the top plate, and the weak intrinsic absorption loss in the materials, some dissipation of the edge state is unavoidably observed.

Next, we fabricate an amorphous PTI with DI = 0.1 and characterize it using the same experimental setup. In bulk transmission measurements, we observe a significant dip in both forward and backward transmission between 10.6 GHz and 11.4 GHz, indicating a mobility gap at frequencies close to those of the crystalline counterpart (Fig. 2d). In edge measurements, we observe a very large difference between forward and backward transmission in the frequency range of the mobility gap (Fig. 2d). Mapping out the field distributions reveals a unidirectional edge state propagating clockwisely. These experimental results are consistent with numerical calculations (see Fig. 1d), indicating that the localization length is extremely short from ~10.5 GHz to ~11.5 GHz. Since the amorphous PTI lacks periodicity, it lacks a properly defined momentum-space band structure; to characterize the topology, we adapt the Bott index (see Supplementary Information Note 3), which acts like the Chern number but can be applied in real space^33,35^. As shown in Fig. 2a, the Bott index has a nontrivial value of 1 (equivalent to the Chern number for the earlier crystalline PTI) within the mobility gap. All of these results—the bulk gap, one-way chiral edge transport, first-principles calculations of the mobility gap, and the Bott index—are in excellent agreement, pointing to the existence of topologically protected edge states in the amorphous PTI.

**Fig. 3:**
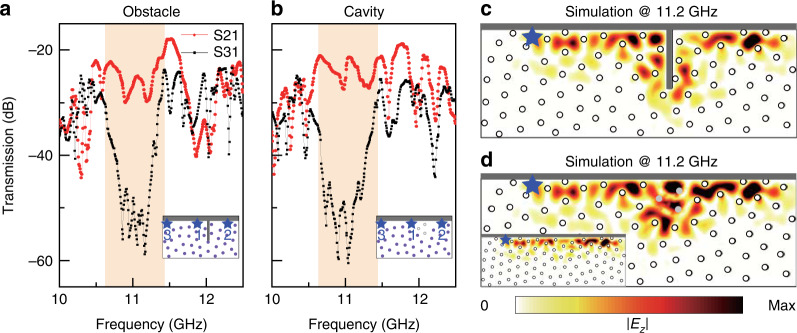
Robust chiral edge propagation in an amorphous PTI with defects. **a** Measured edge transmission in the presence of a large obstacle. Inset: schematic of the experimental setup, where the source (1) and probe (2 or 3) antennas are marked as stars. Gray bars represent the metallic obstacle and boundary. The length of the obstacle is 3*a*. **b** Measured edge transmission in the presence of a large cavity. Inset: schematic of the experimental setup. The light gray dots denote three removed gyromagnetic rods. **c**, **d** Simulated |*E*_*z*_| field distribution in the presence of a large obstacle and a large cavity, respectively. Inset: simulated |*E*_*z*_| field distribution without defects. The star denotes the point source

To verify the robustness of the edge states in the amorphous PTI, we introduce defects along the edges. Two types of defects are tested. In the first case, a rectangular copper obstacle is placed at the edge to block edge propagation (Fig. 3a). In the second case, three gyromagnetic rods are removed to create a large air cavity (Fig. 3b). We then measure the edge transmission. In both cases, we find large differences between forward and backward transmission in the frequency range of the mobility gap, indicating that the defects do not cause backscattering. In addition, we also perform a numerical simulation to further confirm the unidirectional edge states in the presence of such strong defects. One can see that the edge state travels around the obstacle (Fig. 3c) or through the cavity (Fig. 3d). All these results verify that the topological edge states in amorphous PTIs are immune to backscattering and robust against defects. We also numerically show that the topological edge states are robust not only to position disorder but also to disorder in the size of the rods in the Supplementary Information Note 4.

**Fig. 4:**
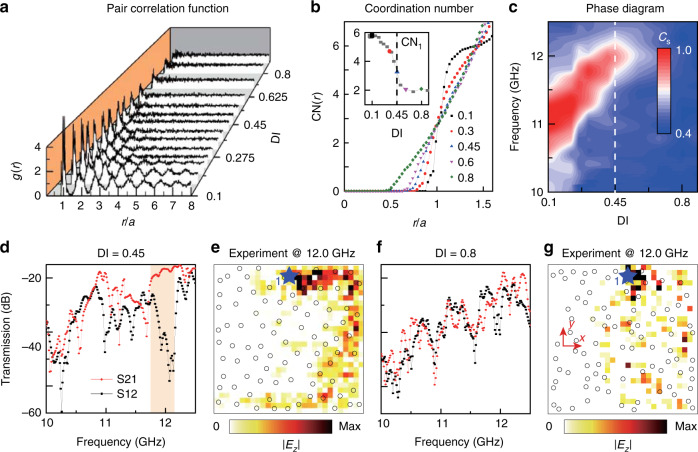
Extinction of topological states in photonic amorphous lattices. **a** Pair correlation function with varying DI. **b** Running coordination number of photonic lattices with DI = 0.1–0.8. Inset: coordination numbers of the first pair correlation function peak (CN_1_). **c** Numerically calculated empirical parameter *C*_s_. Dotted lines (black in **b** and white in **c**) represent the critical DI approaching the short-range order threshold. **d**–**g** Measured transmission and |*E*_*z*_| field distribution of the edge states in photonic lattice samples with DI = 0.45 and 0.8, respectively. The experimental setup is the same as that shown in Fig. 2. The orange region in **d** denotes the corresponding numerically calculated topological region

Next, we study the effects of the glass transition. It should be noted that the nature of the glass transition in amorphous materials remains poorly understood, despite extensive theoretical and experimental studies^36,37^. Using the lattice generation procedure detailed above, Fig. 4a plots how the pair correlation function evolves with the DI. To help locate the glass transition, we calculate the running coordination number

2 $${\mathrm{CN}}(r) = \frac{{2\pi N}}{{L^2}}{\int_0^r} {{g(r)r{\mathrm{d}}r}}$$

As shown in Fig. 4b, CN(*r*) changes from a step-like curve to a smooth curve with increasing DI. Integrating to the first minimum (a discontinuity) of *g*(*r*) gives the coordination number of the nearest neighbors^48^, denoted as CN_1_ (inset of Fig. 4b), which represents the average local connectivity of each lattice site. As the DI increases, CN_1_ decreases from a value of ~6 (similar to the crystalline case) to ~2 (similar to a liquid). Around the critical value of DI = 0.45, CN_1_ decreases very quickly, suggesting a glass transition^36,48^. Thereafter, CN_1_ converges to ~2, indicating the completion of the glass transition.

We used first-principles simulations to investigate the interplay between short-range order and topological protection. In the simulations, the photonic lattices are surrounded by PEC boundaries, and a point source is placed near the boundaries. Based on the numerical field distributions, we calculate an empirical parameter^32^

3 $$C_{\mathrm{s}} = \frac{{{\int}_{{\Pi}_{\mathrm{s}}} {\varepsilon (x,y){\mathrm{d}}x{\mathrm{d}}y} }}{{{\int}_{\Pi} {\varepsilon (x,y){\mathrm{d}}x{\mathrm{d}}y} }}$$

where *ε* is the electromagnetic (EM) energy density, Π is the whole area of the photonic lattice, and Π_s_ is the area one free space wavelength away from the PEC boundary. When the system hosts topological edge states, they tend to be localized in Π_s_, so *C*_s_ is close to unity. A plot of *C*_s_ versus the DI is shown in Fig. 4c. For DI = 0.1, *C*_s_ is close to unity within the frequency window corresponding to the mobility gap, consistent with the previous results. Upon increasing the DI above the critical value of 0.45, i.e., near the glass transition, the short-range order quickly decreases, and the frequency window (the high *C*_s_ region marked in red in Fig. 4c) shrinks rapidly to zero.

To verify these findings experimentally, we fabricate two samples with DI = 0.45 and DI = 0.8 and measure the edge transmission and the electric field distribution using the same setup shown in Fig. 2. For DI = 0.45, the topological frequency window shrinks to a narrow range of 11.7–12.2 GHz (Fig. 4d), and the edge states are only weakly confined to the edge (Fig. 4e). For DI = 0.8, past the glass transition, there is no sign of topological edge states in the transmission or field distribution measurements; the numerically calculated localization length shows small fluctuations (Fig. 1d), suggesting the closing of the mobility gap.

## Discussion

We thus experimentally realized amorphous PTIs that lack long-range order but preserve short-range order. Using microwave measurements, we directly observed the bulk mobility gap and the unidirectional propagation of topological edge states, which is robust against defects and disorder. By gradually deforming the amorphous lattice into a liquid-like lattice through the glass transition, we observed the closing of the mobility gap and the disappearance of the topological edge states. These results illustrate the key role of short-range order in the formation of topological edge states. These insights may be useful for realizing amorphous topological insulators in other physical settings, such as acoustics. It would also be interesting to explore other types of non-crystalline photonic topological materials, such as topological random lasers.

## Materials and methods

### Sample and experimental measurement

The yttrium iron garnet (YIG) ferrite cylinder rods have a relative permittivity of 13, dielectric loss tangent of 0.0002, radius of 2.2 mm, and height of 4 mm. The saturation magnetization was measured to be 4π*M*_*s*_ = 1780 Gauss, and the gyromagnetic resonance loss width was 35 Oe. In the microwave measurements, a static magnetic field generated by an electromagnet is applied perpendicular to the waveguide, producing a strong gyromagnetic response in the ferrite rods. The spatial non-uniformity of the magnetic field is less than 2% in the sample region.

### Simulation

The band structure, bulk/edge transmissions, and field distributions are simulated using the finite element software COMSOL Multiphysics. The relative magnetic permeability of the YIG has the form

$$\tilde \mu = \left[ {\begin{array}{*{20}{c}} {\mu _{\mathrm{r}}} & {i\kappa } & 0 \\ { - i\kappa } & {\mu _{\mathrm{r}}} & 0 \\ 0 & 0 & 1 \end{array}} \right],$$

where $\mu _{\mathrm{r}} = 1 + \frac{{\left( {\omega _0 \,+\, i\alpha \omega } \right)\omega _m}}{{\left( {\omega _0 \,+\, i\alpha \omega } \right)^2\,-\,\omega ^2}}$, $\kappa = \frac{{\omega \omega _m}}{{\left( {\omega _0 \,+\, i\alpha \omega } \right)^2 \,-\, \omega ^2}}$, $\omega _m = \gamma \mu _0M_s$, $\omega _0 = \gamma \mu _0H_0$, *μ*_0_*H*_0_ = 0.2 T is the external magnetic field along the −*z* direction, *γ* = 1.76 × 10^11^ C kg^−1^ is the gyromagnetic ratio, *μ*_0_ is the permeability of free space, *α* = 0.0088 is the damping coefficient, and *ω* is the operating frequency.

## Supplementary information

Supplementary Information

## Data Availability

The data that support the plots presented in this paper and other findings of this study are available from the corresponding author upon request. Supplementary information accompanies the manuscript on the Light: Science and Applications website (http://www.nature.com/lsa).
