# Supplementary material for: Photonic amorphous topological insulator
Source: Light Sci Appl. 2020 Jul 24;9:133. doi: 10.1038/s41377-020-00368-7 (PMC7381680; doi:10.1038/s41377-020-00368-7)
Supplement: Supplementary file 1 — Supplementary Information [file 41377_2020_368_MOESM1_ESM.docx]

Supplementary Information for

**Photonic amorphous topological insulator**

Peiheng Zhou^1,#^, Gui-Geng Liu^2,#^, Xin Ren^1^, Yihao Yang^2,3,*^, Haoran Xue^2^, Lei Bi^1^, Longjiang Deng^1^, Yidong Chong^2,3,*^, and Baile Zhang^2,3,*^

^1^National Engineering Research Center of Electromagnetic Radiation Control Materials, University of Electronic Science and Technology of China, Chengdu 610054, China.

^2^Division of Physics and Applied Physics, School of Physical and Mathematical Sciences, Nanyang Technological University, 21 Nanyang Link, Singapore 637371, Singapore.

^3^Centre for Disruptive Photonic Technologies, The Photonics Institute, Nanyang Technological University, 50 Nanyang Avenue, Singapore 639798, Singapore.

^#^These authors contributed equally to this work.

**Supplementary Information Note 1: Generation of disordered photonic lattices**

The disordered photonic lattices are generated using an event-driven molecular dynamics procedure proposed in Ref. [1]. In a 2D square unit cell, 576 discs are periodically filled in the Euclidean space; half of the discs have radii 1.2 times the other half. Periodic boundary conditions are applied to eliminate the effects of the computational cell walls. The packing process starts with a Poisson distribution of points; the points then grow into nonoverlapping discs. During the growth, the discs move according to Newtonian mechanics including energy non-conserving collision events. The growth rate is always positive, and needs to be handled carefully; to generate the amorphous lattices with high levels of correlation, it needs to be fast enough to suppress crystallization.

Particle patterns with DI = 0.1, 0.45 and 0.8 are shown in Fig. 1**a** in the main text. These packing patterns are expanded from the original 2D square cell to an implementation size of 24*a* × 24*a*, where *a* = 17.5 mm is the characteristic length defined in the main text. Due to the initial bidispersivity, two kinds of discs with radius ratio 1.2 are randomly distributed in each pattern. Since the nearest neighbour (NN) distance is decided by the expanded radii of the two kinds of discs, there are three kinds of NN distance: the distance between two neighbouring small discs, between one big and one small discs, and between two big discs. The ratio of these three distances is 1:1.1:1.2 due to the chosen radius ratio. For comparison, for the ideal triangular particle packing pattern (DI = 0 in Fig. 1**a** in the main text), each disc has 6 NNs of the same radius. Since the densest packing is *ϕ*_max_ = 0.9069, the NN distance is the diameter of the discs, $d_{\text{triangular}}=48a\sqrt{\frac{\varphi_{\max}}{\pi N}}$, where *N* = 576 is the number of discs in the system. We find that *d*_triangular_ = 18.81 mm for the chosen parameters.

Finally, the discs are replaced by gyromagnetic rods with radius *r* = 2.2 mm, resulting in the photonic lattices shown in Figs. S1**a**-**c**. For the ideal triangular lattice, the lattice constant is equal to the NN distance, says *a*_c_ = *d*_triangular_ = 18.81 mm. The photonic lattices are truncated to dimensions 9*a* × 9*a*, which accommodate 85 rods. The density of rods is a constant in all samples. Note that a full-sized 24*a* × 24*a* lattice is employed for the calculation of pair correlation function (Fig. 1**c** of the main text). The truncated lattices shown in Figs. S1**d**-**f** are the same as those employed for experiments in the main text.


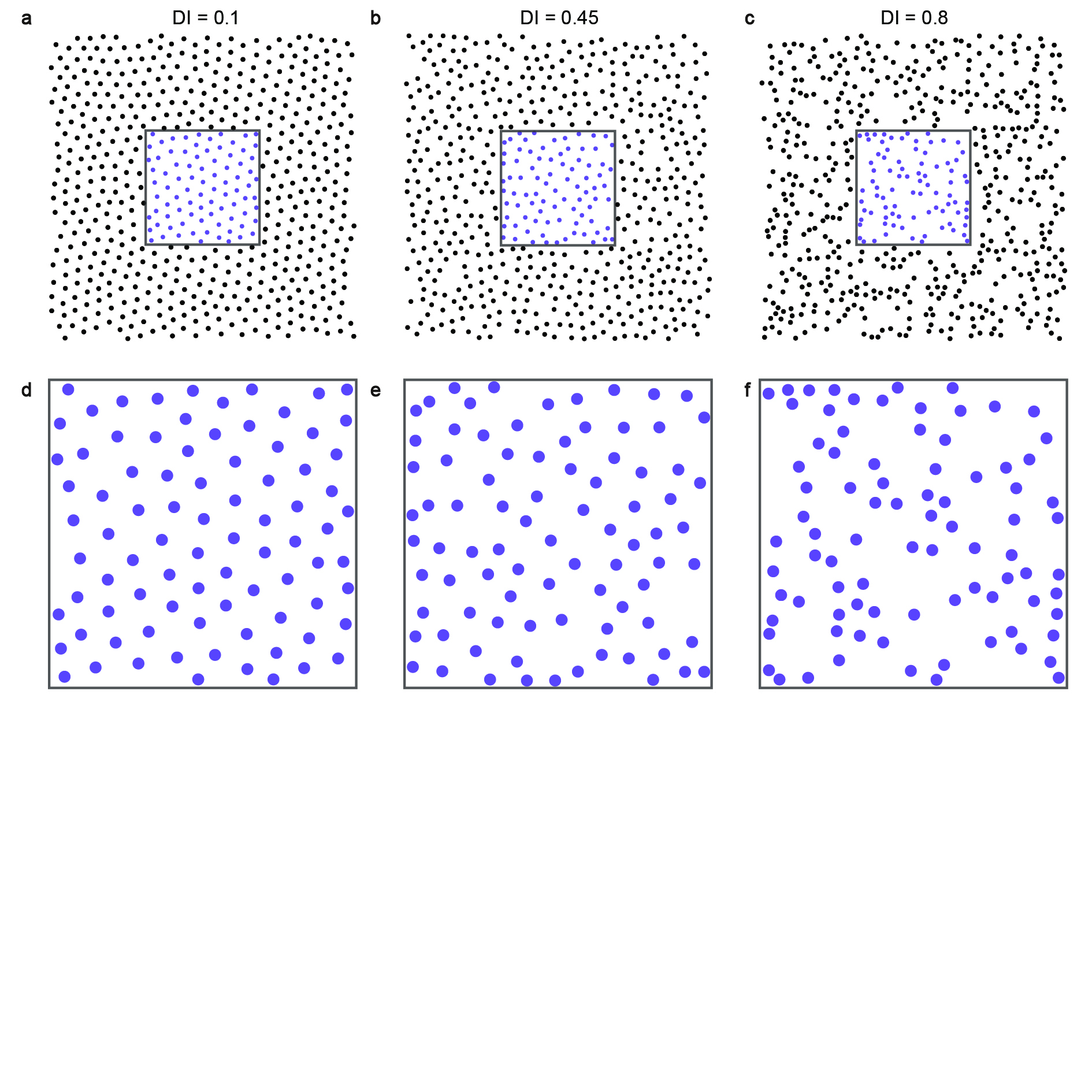


**Fig. S1 Configurations of the photonic lattices. a**-**c** Disordered photonic lattices generated via packing. Gray squares indicate the truncation areas. **d**-**f** Close-up views of the truncated photonic lattice configurations used in the experiments.

**Supplementary Information Note 2:** **Measurement setup**

To form the parallel plate waveguide (Fig. 2**b** in the main text), ferrite rods are fixed to the bottom copper plate. It is important for both the top plate and the bottom plate to have good contact with the ferrite rods. As a modification of the classical waveguide design shown in the top panel of Fig. S2**a**, we drill a square array of cylindrical holes through the top copper plate (bottom panel of Fig. S2**a**). These holes have radius 1mm and center-to-center distance 5.62 mm, and serve as antenna ports in the microwave measurements. This provides great flexibility for placing the source and probe at different positions within the area of the sample without moving the waveguide assembly.


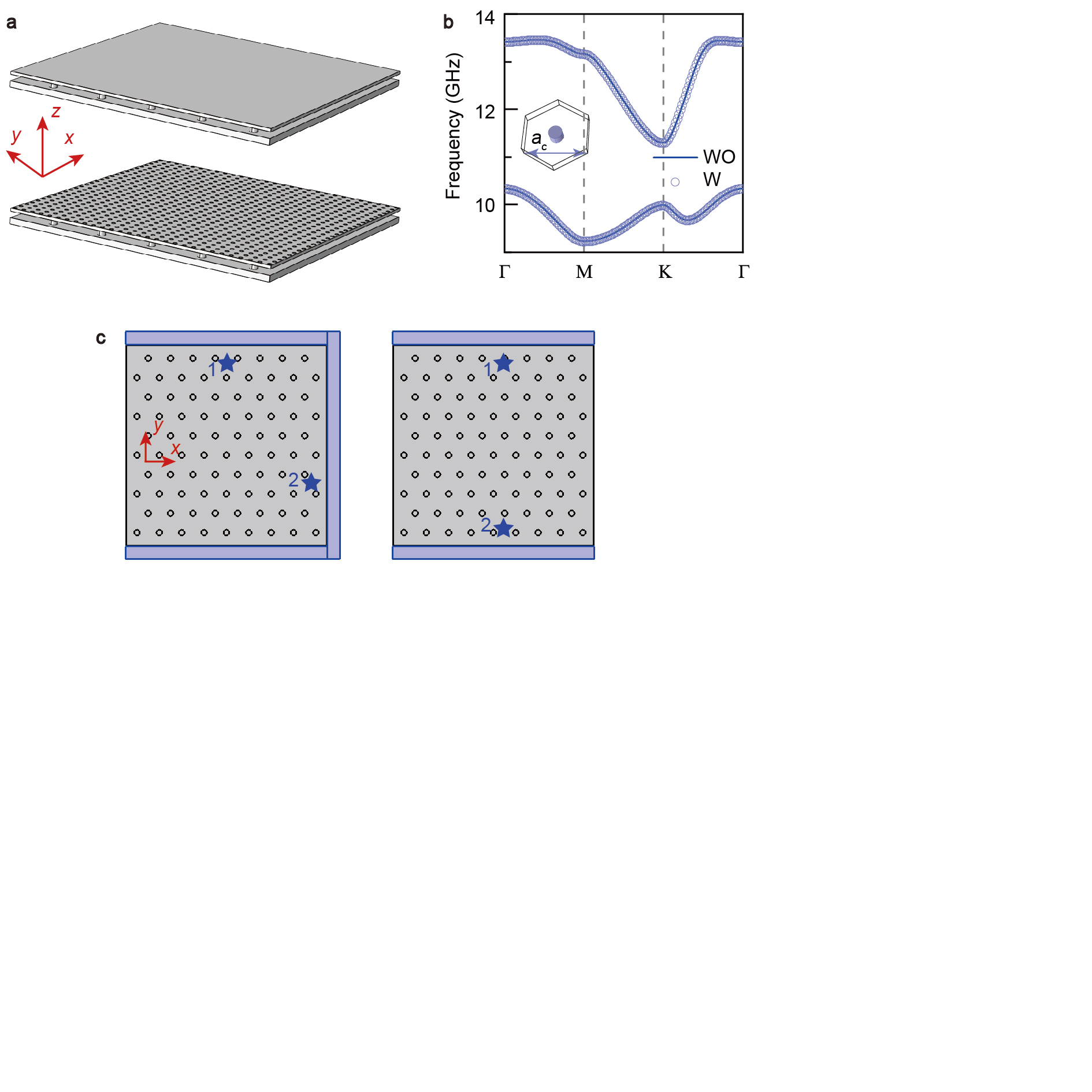


**Fig. S2 Waveguide setup and the effect of top plate design. a** Waveguide configurations for the experiments and simulations. The top panel shows the classical setup using a metallic sheet as top plate. The bottom panel shows the actual setup, which has a square array of holes in the top plate. **b** Dispersion relation for the two types of waveguide. Inset: unit cell of the triangular lattice inside the waveguide. The gyromagnetic rod is placed at the center of the unit cell. The lattice constant is *a*_c_ = 18.81 mm. Blue lines indicate the classical waveguide (top plate without holes) (WO). Blue circles indicate the waveguide with holes in the top plate (W). Only the second and third bands are shown. **c** Configuration of the waveguide setup for edge transmission (left panel) and bulk transmission (right panel) measurement. The top plate is removed to show the inside arrangement in the waveguide. The source antenna (1) and the probe antenna (2) are marked by blue stars. The blue bars represent the perfect electric conductors.

We performed a numerical study of the eigenmodes of the waveguide. Figure S2**b** shows the resulting dispersion relations for both the classical waveguide without holes in the top plate (WO) and with holes in the top plate (W). In both cases, a triangular lattice photonic crystal is loaded in the waveguide (inset of Fig. S2**b**). In these simulations, the metal regions are modeled as PECs. Other lateral boundaries have periodic boundary conditions. We find that the air holes have negligible effect on the dispersion relation, as is to be expected since they are much smaller than the operating wavelength. However, in microwave measurements, there are still small radiation losses due to these holes.

For the bulk and edge transmission measurements, different types of boundaries are employed as shown in Fig. S2**c**. For edge state measurements, three PEC boundaries wrap the waveguide clock-wisely, with microwave absorber on the remaining boundary. The source antenna and probe antenna are separated by a 90 º corner to measure the unidirectional robust propagation of topological edge states. For bulk state measurements, two PEC boundaries are placed at opposite sides of the sample, with absorbers on the other two boundaries. The source antenna and probe antenna are located close to each PEC boundary.

**Supplementary Information Note 3: Calculation of Bott index, *C*_s_, and *l*_loc_**


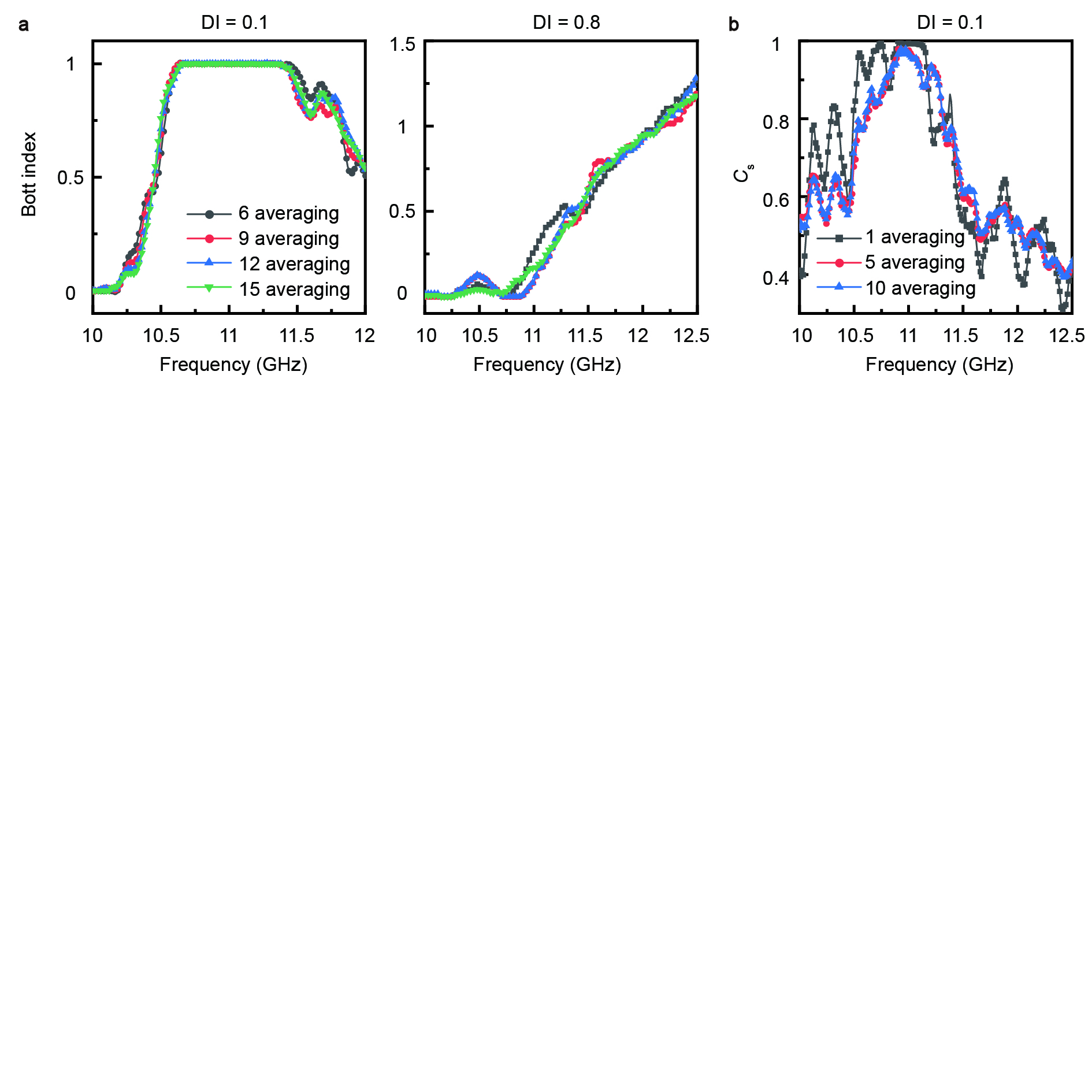


**Fig. S3 Calculated parameters of the amorphous PTI vs realization number. a** Configuration averaged Bott index for both DI = 0.1 and 0.8. The averaging of the calculated results is conducted at different realization numbers, from 3 realizations to 15 realizations. **b** Configuration averaged *C*_s_.

The Bott index is calculated in real space by applying periodic boundary conditions to the amorphous lattices [2, 3]. In a 2D lattice of *L*_1_ × *L*_2_ lattice sites, we define the operatorsand ,where (*l*_1_, *l*_2_) is the lattice site. These two operators are used to find the band-projected position matrices on a torus to describe our topological invariant. Let the projection operator be given by

$$\hat{P}=\sum_{j=1}^{N} |\psi_{j}\rangle\langle\psi{}_{j}|$$

with $|\psi_{j}\rangle$ (*j* = 1, …, *N*) the eigen states of the Hamiltonian. Then, we can calculate the block matrices and, which are almost commute and unitary. One can find this by noting that $\hat{P}$ is almost commute with both $\hat{U}$and $\hat{V}$, as $\hat{P}$ is local in an energy (frequency) gap. Then the Bott index is given by

.

Due to the approximating community and unitarity of $\tilde{U}$ and $\tilde{V}$, the quantity

has real *r* and real integer *C*_b_ since . The trivial value *C*_b_ = 0 can be obtained for exactly commute block matrices.

To obtain the Bott index in the amorphous PTIs (DI = 0.1 and 0.8), 15 realizations of the amorphous lattices at the same DI are employed for averaging. Large lattice sizes of 18*a* × 18*a* are truncated. Figure S3**a** shows the effect of averaging at different realization numbers. The calculated results converge with the increase of averaging numbers. For the trivial lattice (DI = 0.8), no mobility gap of integer value 1 can be found. It is worth mentioning that the calculated Bott index rises beyond one, because there is another topological mobility gap around 13.5 GHz whose Bott index is larger than one. This mobility gap corresponds to the bandgap around 14 GHz in the crystalline PTI (see the bandgap in Fig. 2**a** in the main text), which exhibits a large Chern number. The discussions on large Chern numbers are complex even in crystalline PTIs [4, 5]. Our study focuses on the lowest topological mobility gap, and thus has excluded the discussion of all higher mobility gaps.

For the calculation of *C*_s_, the amorphous PTI lattices are truncated to 16*a* × 16*a* and wrapped by PEC edges using the same setup as Fig. 4**c** of the main text. As shown in Fig. S3**b**, the calculation converges so quickly that the averaging result over 5 realizations is close to the one over 10 realizations. Increase of the realization number smooths the spectral fluctuation.

In a 2D system, the quantum states are localized in the presence of disorder. To determine the localization lengths, we simulate the photonic lattices in a ribbon-like configuration of sizes 16*a* × 48*a*. Periodic boundary conditions are applied on the horizontal edges (perpendicular to the thickness direction) and waveguide port boundaries (one inlet, one outlet, and with the distance *L*_s_ = 48*a* along this propagation direction) are set at the other two boundaries. The localization length *l*_loc_ is defined as [6]

$$l_{\text{loc}}^{-1}=-\lim_{L_{\text{s}}\to\infty} \left\langle\frac{\ln\left|T\right|}{L_{\text{s}}}\right\rangle$$

where *T* is the simulated transmission coefficient. All calculation results are averaged over 18 configurations. As shown by the unnormalized value of *l*_loc_ in Fig. S4, the value of localization reaches a minimum inside the topological region for DI = 0, 0.1, 0.3, and 0.45. For DI = 0, *l*_loc_ < *a* is found inside the 10.3 GHz - 11.3 GHz region, indicating a full photonic bandgap [7]. For DI = 0.1, we find *l*_loc_ ~*a* inside the 10.7 GHz - 11.4 GHz region, indicating a mobility gap [7]. The mobility gap shrinks and *l*_loc_ increases with the increasing DI, due to the formation of localized states inside the gap region [8]. For DI = 0.8, no clear gap is observed and *l*_loc_ > *L*_s_ at high frequencies. This suggests that short-range order is responsible for supporting the mobility gap.


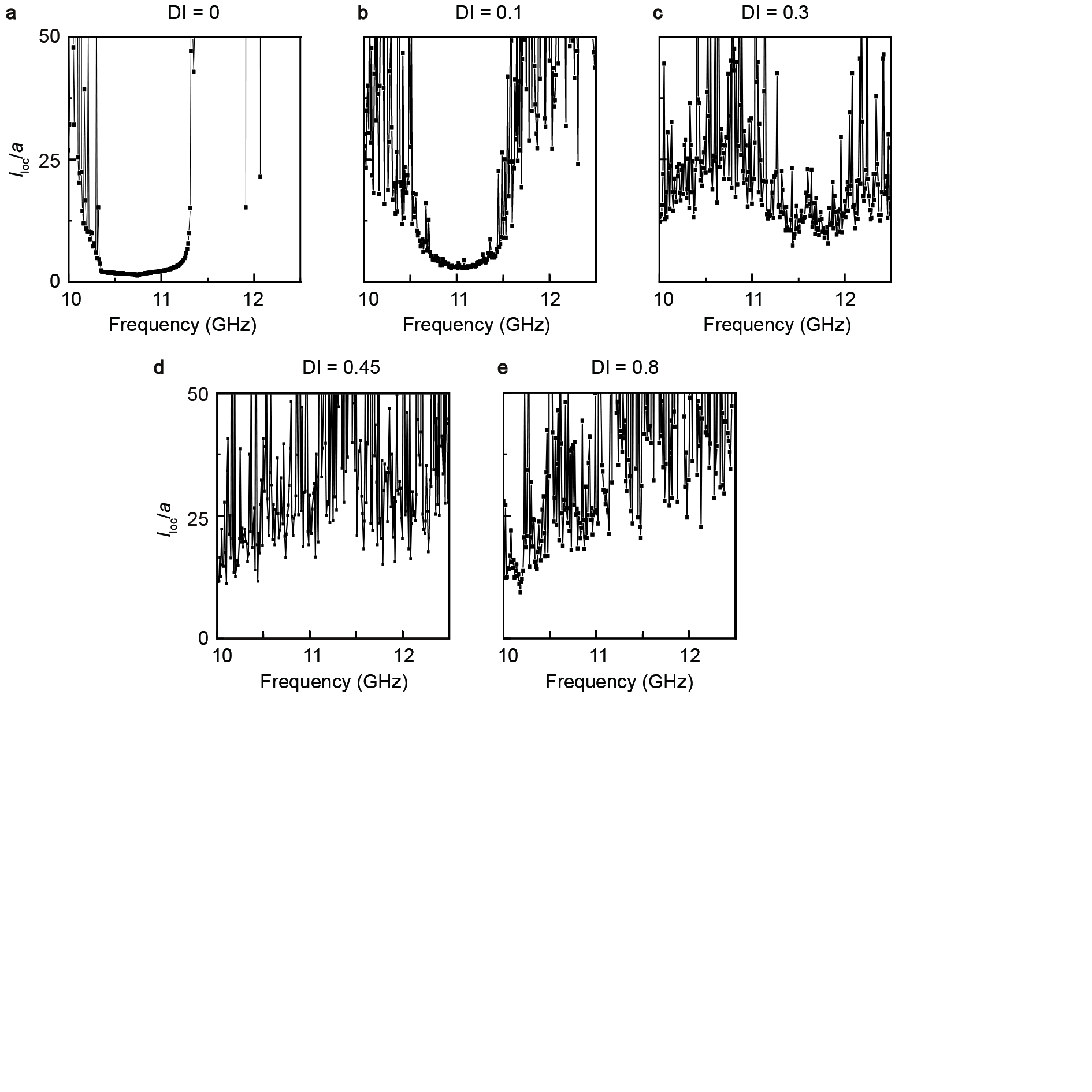


**Fig. S4 Localization lengths in the photonic lattices.** Five values of the DI, ranging from 0 to 0.8, are selected for simulation and calculation. The calculation results of each DI are averaged over 18 realizations.

In the main text, we have also shown the simulated results of bulk transmissions in Fig. 1**d**, which are consistent to the localization analysis and make a clear observation of frequency gap transition. The simulations are conducted in the photonic lattices of sizes 20*a* × 20*a*. Periodic boundary conditions and waveguide port boundaries are imposed to the horizontal and vertical boundaries, respectively. The obtained transmission coefficients are averaged over 18 configurations.

**Supplementary Information Note 4: Disorder in size for amorphous PTIs**


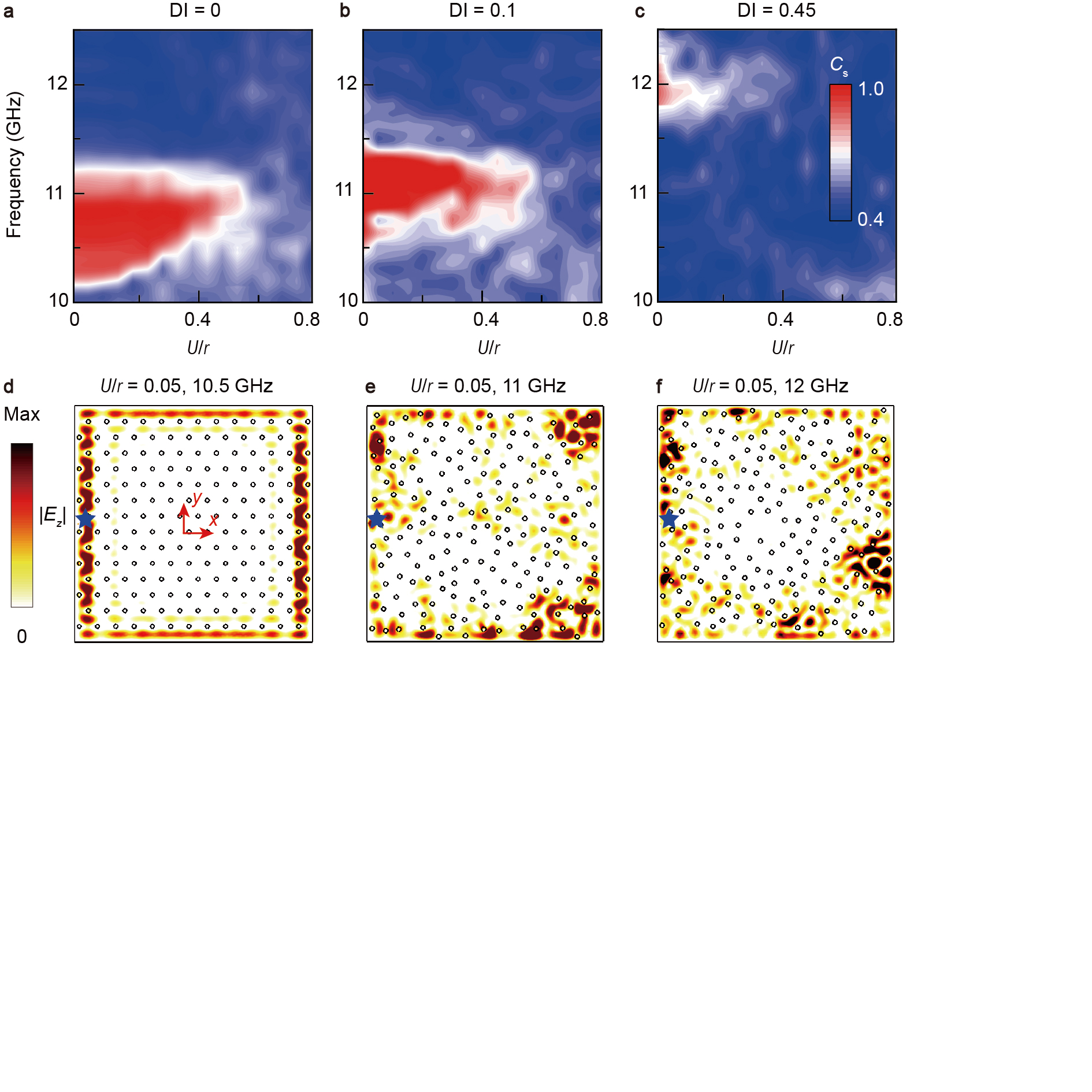


**Fig. S5 Disorder in element radius for the amorphous PTIs. a-c** Numerically calculated empirical parameter *C*_s_. Amorphous PTIs (DI = 0.1 and 0.45) are compared with the crystalline photonic lattice (DI = 0). **d-f** Simulated |*E_z_*| field distribution for the edge states. Each lattice contains 182 gyromagnetic rods in a system (14*a* × 14*a* in size) wrapped by perfect electric conductors. The out-of-plane point sources are indicated by blue stars. Methods used in the simulations and calculations of *C*_s_ are similar to the ones employed for Fig. 4**c** in the main text.

To further inspect the robustness of the chiral edge states in our amorphous PTIs, we introduce fluctuations in the rod radii by setting *r’* = *r* + *UM*, where *r* is the original rod radius, *M* is the random variable distributed uniformly over the interval [-1, 1], and *U* is a positive number representing the disorder strength. As shown in Figs. S5**a**-**c**, the corresponding crucial parameter *C*_s_ reveals a topological frequency window (i.e., the red region) that closes as the disorder strength *U* increases. It is evident that the topological mobility gap also depends on the structural correlation, or the strength of short-range order: the strongly correlated amorphous PTI (DI = 0.1) hosts the topological frequency window up to *U* ~0.6*r*, which is similar to the counterpart in the crystalline situation (DI = 0); the weakly correlated one (DI = 0.45) closes the frequency window at a small value of *U* ~0.1*r*. In Figs.S5**d**-**f**, we plot the field distribution for the cases of DI = 0, 0.1, and 0.45, respectively, all with *U* = 0.05*r*. It can be seen that the edge states still exist when including disorder in size.

**References**

[1] Donev, A., Torquato, S. & Stillinger, F.H. Neighbor list collision-driven molecular dynamics simulationfor nonspherical hard particles. I. Algorithmic details. *J. Comput. Phys.* **202**, 737-764 (2005).

[2] Loring, T.A. & Hasting, M.B. Disordered topological insulators via C*-algebras. *Europhys. Lett.* **92**, 67004(2010).

[3] Ge, Y. & Rigol, M. Topological phase transitions in finite-size periodically driven translationally invariant systems. *Phys. Rev. A* **96**, 023610(2017).

[4] Skirlo, S.A., Lu, L. & Soljačić, M. Multimode one-way waveguides of large Chern number, *Phys. Rev. Lett.* **113**, 113904 (2014).

[5] Skirlo, S.A., Lu, L., Igarashi, Y., Yan, Q.H., Joannopoulos, J. & Soljačić, M. Experimental observation of large Chern numbers in photonic crystals, *Phys. Rev. Lett.* **115**, 253901 (2015).

[6] Asatryan, A.A., Botten, L.C., Byrne, M.A., Freilikher, V.D., Gredeskul, S.A., Shadrivov, I.V., McPhedran, R.C. & Kivshar, Y.S. Suppression of Anderson localization in disordered metamaterials, *Phys. Rev. Lett.* **99**, 193902 (2007).

[7] Froufe-Pérez, L.S., Engel, M., José Sáenz, J. & Scheffold, F. Band gap formation and Anderson localization in disordered photonic materials with structural correlations. *Proc. Natl. Acad. Sci. USA* **114**, 9570-9574 (2017).

[8] Sigalas, M.M., Soukoulis, C.M., Chan, C.-T. & Turner, D. Localization of electromagnetic waves in two-dimensional disordered system. *Phys. Rev. B* **53**, 8340-8348 (1996).
